# Treat to target: temporomandibular joint (TMJ) arthritis in children with juvenile idiopathic arthritis (JIA) – experience of Centro Hospitalar Do Porto

**DOI:** 10.1186/1546-0096-12-S1-P188

**Published:** 2014-09-17

**Authors:** Joao Nascimento, Carla Zilhão, Asdrubal Pinto, Carlos Miranda, Carlos Monteiro, Margarida Guedes

**Affiliations:** 1 Pediatric Rheumatology Unit, Centro Hospitalar Porto, Porto, Portugal; 2 Oral-Maxillofacial Surgery, Centro Hospitalar Porto, Porto, Portugal

## Introduction

TMJ arthritis occurs in up to 80% of children with JIA and it has been described in all JIA subtypes. TMJ arthritis can develop anytime during the course of JIA, even when patients are asymptomatic or on biologic therapy. As it can also occur in the absence of clinically evident arthritis outside the TMJ, a precocious evaluation by maxillofacial surgery is suggested.

## Objectives

Evaluation of TMJ in JIA and presentation of adopted guidelines in Centro Hospitalar do Porto.

## Methods

Selection of three polyarticular JIA cases with TMJ involvement followed in pediatric rheumatology unit. The protocol establishes oral-maxillofacial observation and high-resolution ultrasound at clinical presentation of polyarticular JIA. The need of contrast-enhanced magnetic resonance imaging, the periodicity of clinical observations and the decision to perform intraarticular corticosteroids injections is based on the clinical repercussion of TMJ arthritis.

## Results

### Case 1

15 year old girl, with polyarticular JIA diagnosed in 2012 and on etanercept during the past 14 months. The TMJ response to biologic therapy was minimal and maximal incisional opening (MIO) was 37mm.Intraarticular corticosteroid injection (triamcinolone hexacetonide -1ml; 20mg/ml) was performed and three months after arthroscopy, pain relief was achieved with a good MIO improvement.

### Case 2

11 year old girl, with psoriatic JIA diagnosed in 2008, on etanercept during the past 22 months and intolerant to methotrexat. She has micrognathia, malocclusion of the teeth with overbite (class 2) and a painful TMJ. Condylar flattening was observed in the last magnetic resonance imaging.

### Case 3

14 year old girl, with polyarticular JIA diagnosed in 2012 and on etanercept during the past 12 months. Facial asymmetry and sporadic pain when opening the mouth were present. TM joint dysfunction with limited left condylar excursion was confirmed in the last high-resolution ultrasound.

## Conclusions

The TMJ involvement in JIA, presented in the cases, was responsible for the recent creation of a multidisciplinary protocol joining pediatric rheumatology and oral-maxillofacial surgery of Centro Hospitalar do Porto. The main goal is to guarantee a precocious TMJ examination, with early treatment when necessary, to avoid some deleterious consequences that develop even in the absence of symptoms.

## Disclosure of interest

None declared.

